# Parity and Metabolic Syndrome Risk: A Systematic Review and Meta-Analysis of 15 Observational Studies With 62,095 Women

**DOI:** 10.3389/fmed.2022.926944

**Published:** 2022-07-12

**Authors:** Ming-Hui Sun, Zhao-Yan Wen, Ran Wang, Chang Gao, Jia-Li Yin, Yu-Jiao Chang, Qi-Jun Wu, Yu-Hong Zhao

**Affiliations:** ^1^Department of Clinical Epidemiology, Shengjing Hospital of China Medical University, Shenyang, China; ^2^Clinical Research Center, Shengjing Hospital of China Medical University, Shenyang, China; ^3^Key Laboratory of Precision Medical Research on Major Chronic Disease, Shengjing Hospital of China Medical University, Shenyang, China; ^4^Department of Obstetrics and Gynecology, Shengjing Hospital of China Medical University, Shenyang, China

**Keywords:** metabolic syndrome, meta-analysis, observational study, parity, systematic review

## Abstract

**Background:**

Epidemiological studies have provided inconsistent evidence of the association between parity and metabolic syndrome (MetS) risk. We conducted this first systematic review and meta-analysis to comprehensively and precisely quantify this topic.

**Methods:**

Comprehensive searches of PubMed, Embase, and the Web of Science databases were conducted to identify observational studies of the association between parity and MetS risk up to 30 January 2022. Study inclusion, data extraction, and quality assessment were checked and reviewed by two investigators independently. Random-effects models were applied to estimate pooled odds ratios (ORs) and 95% CIs. This study has been registered with PROSPERO.

**Results:**

Two high-quality cohorts and thirteen medium-quality cross-sectional studies involving 62,095 women were finally included. Compared with the nulliparous, the pooled OR of MetS for the ever parity was 1.31 (95% CI = 0.91–1.88, *I*^2^ = 72.6%, *n* = 3). Compared with the lowest parity number, the pooled OR of MetS for the highest parity number was 1.38 (95% CI = 1.22–1.57, *I*^2^ = 60.7%, *n* = 12). For the dose-response analysis, the pooled OR of MetS for each increment of one live birth was 1.12 (95% CI = 1.05–1.19, *I*^2^ = 78.6%, *n* = 6). These findings were robust across subgroups and sensitivity analyses. No evidence of heterogeneity between subgroups was indicated by meta-regression analyses.

**Conclusion:**

The findings suggested that parity was associated with an increased risk of MetS. A sufficient number of large prospective cohort studies are required to fully verify our findings.

**Systematic Review Registration:**

[https://www.crd.york.ac.uk/PROSPERO/], identifier [CRD42022307703].

## Introduction

Metabolic syndrome (MetS) is a group of clinical syndromes, including abdominal obesity, hypertension, hyperglycemia, hyperlipidemia, and low high-density lipoprotein cholesterol levels (1). The impacts of MetS are wide-reaching, including an increased risk of diabetes, cardiovascular disease, various cancers, and all-cause mortality (2–4). It is estimated that approximately one-quarter of adults worldwide suffers from MetS (1). In 2016, a review of 35 articles, including 226,653 Chinese subjects, indicated that the prevalence of MetS in the Chinese adult population was about 24.5% (5). The prevalence of MetS has been on the rise in recent years. From 1999 to 2014, the prevalence of MetS in the United States population increased from 27.9 to 31.5% (6). Beside genetic and environmental factors (7, 8), epidemiological studies have shown that reproductive factors may play an important role in the development of MetS (9–11).

Pregnancy can trigger a series of changes in estrogen levels and metabolic systems in women (12). While these changes occurred during pregnancy and can be reversed after delivery, the long-term impacts of the state of physiological changes can result in an increased risk of diabetes and cardiovascular disease among women (12–14). An increased number of pregnancies, at the same time, leads to a lifetime of estrogen reduction and insulin resistance, which leads to an increased risk of MetS in turn (15, 16). Up to now, results regarding the association between parity and MetS are inconsistent. Some investigators have argued that a statistically significant association between parity and MetS risk exists (10, 17), whereas others have argued that parity was not associated with MetS (18–20). For instance, a recent cross-sectional study in 2018 reported a significant positive relationship between parity and MetS risk (21). In contrast, a study in Korea indicated no association between parity and the risk of MetS in parous women after adjusting for potential confounder factors (20).

Given these inconsistent findings, an up-to-date understanding of the association between parity and MetS risk is warranted. Nevertheless, to the best of our knowledge, no published literature comprehensively and quantitatively analyses the aforementioned association. Herein, we carried out this systematic review and meta-analysis to first evaluate the strength and quality of evidence on this topic.

## Methods

### Data Sources and Search Strategy

This study was reported and conducted according to the Preferred Reporting Items for Systematic Reviews and Meta-Analysis (22) and Meta-Analysis of Observational Studies in Epidemiology guidelines (23). Before searching the literature, this study was also registered (24), with the PROSPERO registration number CRD42022307703.

All the relevant literature published in PubMed, Embase, and the Web of Science databases (up to 30 January 2022) were independently searched by two authors (M-HS and Z-YW). The literature search comprised the following keywords: (“parity” or “multiparity” or “live birth” or “pregnancy” or “reproductive” or “reproduction” or “reproductive factor” or “gravidity” or “fertility”) and (“metabolic syndrome” or “insulin resistance syndrome” or “plurimetabolic syndrome” or “Reaven syndrome” or “syndrome X” or “metabolic syndrome X” or “dysmetabolic syndrome X” or “MetS”). In addition, additional relevant articles identified through the list of references included related articles.

### Study Selection

First, the selected citations were imported into reference management software, EndNote version 7.0 (Thomson Corporation, Stanford, CT, United States) for initial screening and the literature was deleted by duplicate titles. Second, the irrelevant research was excluded by title and abstract. Third, the full text of the article that met the inclusion and exclusion criteria was downloaded and reviewed for eligibility.

The above steps were conducted by two authors (M-HS and Z-YW). Any discrepancies in selected studies were resolved by a third author (Q-JW). Literature meeting the following eligibility criteria was selected: (1) observational study (cross–sectional, cohort, and case–control study); (2) parity as exposure; (3) MetS as the outcome; and (4) the study provides estimates with an odds ratio (OR) or relative risk (RR) and 95% CIs or SEs. However, we exclude the following eligibility criteria studies: (1) the study designs were randomized controlled trials, *in vitro* or animal studies, abstracts, reviews, duplicated data, and meta-analyses and (2) the study was not published in the English language.

### Data Extraction and Quality Assessment

Relevant information was independently extracted by M-HS and Z-YW. All the discrepancies in extracted information were solved by discussion and adjudication with a third author (Q-JW), as needed. We extracted the following data: first author, study year, region, study design, study population, categories of parity exposure, effect estimates and 95% CI for all the MetS outcomes associated with parity, and adjustment for covariates. When several effect estimates (with varying inclusion of covariates) were present, we extracted the effect estimates for adjusted to the most confounders. We also extract case and non-case for different studies in the statistical analysis. When we were unable to obtain additional information, we tried to contact the corresponding author.

Two validated tools were conducted to evaluate the study’s quality: the Quality Assessment Tool for cross-sectional and the Newcastle–Ottawa Scale (NOS) tool. The National Institute of Health Quality Assessment Tool evaluates the risk of cross-sectional studies. The tool assesses 11 domains of bias. We classified the overall risk of bias assessment scores <6 were poor, 6–9 was fair, and 10–11 was identified as good quality (25). The NOS tool evaluates the risk of cohort studies (26). The NOS tool assesses eight fields of bias, composed of three domains for selection, group comparability, and outcome.

Included studies that obtained the maximum number of stars in at least two domains were considered good quality (27).

### Statistical Analysis

In the meta-analysis, effect sizes for parity were extracted from original studies and the RR estimate was considered as an approximation of the OR estimate (28). A random-effects model was applied to evaluate the overall OR estimate ever parity vs. nulliparous and highest vs. lowest categories of parity for the association between parity and MetS.

The linear dose-response relationship between parity and the risk of MetS was calculated. The pooled OR and 95% CI for each increment of one live birth were evaluated using the method by Orsini and Greenland (29). Distribution cases and non-cases need to be provided and effect estimates, such as OR or RR and 95% CI for at least three quantitative exposure categories, are known. For studies that presented by ranges, we estimated the midpoint value in each category by calculating the average of the upper and lower boundaries. When the highest category has no upper boundary, we need to assume the width of the category that had the same as the preceding category. When the lower boundary of the lowest category was not presented, we need to assume the lower boundary to be zero. In addition, due to the limited included study (*n* = 3) in the present analyses, therefore, we fail to calculate a non-linear relationship between parity and MetS risk.

The I squared (I^2^) test was used to calculate the heterogeneity of the included research; I^2^ values between >75, 50 to 75%, and <50% were considered to classify high, moderate, and low heterogeneity, respectively (30). Subgroup analyses were conducted for variables, for instance, study location, number of the study population, study design, menopausal status, diagnostic criteria, and adjustment for confounding factors. We use a meta-regression model to evaluate heterogeneity between different subgroups. Sensitivity analyses were conducted by omitting one study every time and calculating the impact of each research on the overall OR to prove the stability of the results. In addition, Egger’s linear regression, Begg’s rank test, and visual inspection of the funnel plot were evaluated in a publication bias (31, 32). We used a trim-and-fill method if possible publication bias was found to analyze the potential effect (33). All the meta-analyses were conducted using Stata software version 12 (StataCorp, College Station, TX, United States).

## Results

### Search Results, Study Characteristics, and Quality Assessment

**FIGURE 1:**
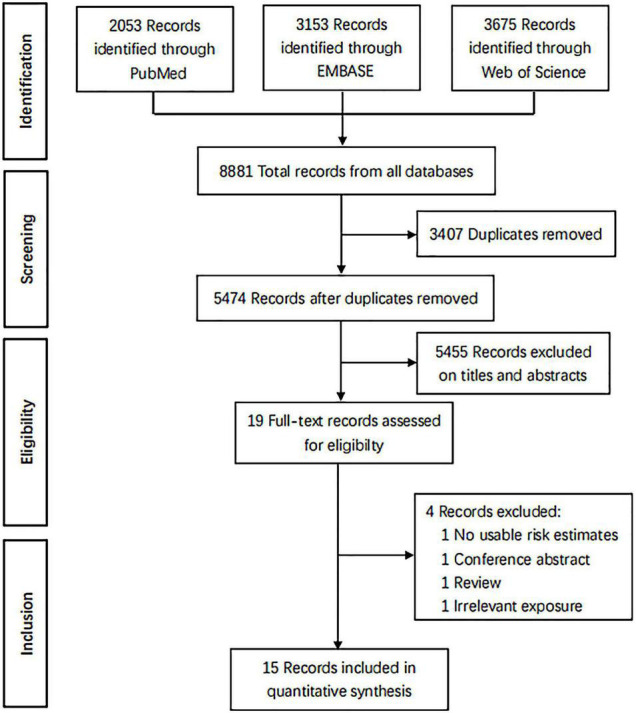
The PRISMA flow diagram.

Through a search from 3 databases, a total of 8,881 records were identified. After 3,407 duplicate records were removed, 5,474 potentially eligible records remained. According to the titles and abstracts, 5,455 records were excluded due to exclusion criteria. Thereafter, the full text of the remaining 19 records was assessed; 4 records were excluded due to no provided risk estimates or 95% CIs, conference abstract, review, and irrelevant exposure (34–37). Finally, 15 records were included for the final analysis (Figure 1).

The principal feature of the 15 articles is shown in Table 1. All of these articles were published between 2000 and 2022, involving 62,095 women. Six of the 15 studies were performed in China (10, 18, 19, 38–40), three studies were conducted in the United States (17, 41, 42), two studies each were performed in Korea (20, 21) and Iran (43, 44), and the remaining two studies were performed in Bangladesh (45) and Oman (46).

**TABLE 1:** Characteristics of studies included in the meta-analysis of parity and metabolic syndrome risk.

References	Country	Study design	No. of study population	Diagnostic criteria for MetS	Exposure categories	Risk estimates (95%CI)
Shi et al. (19)	China	Cross-sectional study	776	Harmonized criteria	2 vs. 1 3 vs. 1 ≥4 vs. 1	1.37 (0.86, 2.16) 1.91 (1.10, 3.32) 1.70 (0.87, 3.33)
Xie et al. (18)	China	Cross-sectional study	6,157	NCEP ATP III	≥2 vs. 1	1.37 (0.89, 2.11)
Yao et al. (10)	China	Cross-sectional study	5,674	NCEP ATP III	2 vs. 1 3 vs. 1	1.39 (1.13, 1.73) 1.50 (1.10, 2.05)
Lee et al. (21)	Korea	Cross-sectional study	4,098	Harmonized criteria	3 vs. 2 4 vs. 2	1.40 (1.11, 1.78) 1.38 (1.07, 1.77)
Vladutiu et al. (17)	United States	Cross-sectional study	7,467	Harmonized criteria	4 vs. 1	1.40 (1.00, 2.00)
Moradi et al. (43)	Iran	Cross-sectional study	978	NCEP ATP III	≥2 vs. 1	1.14 (1.02, 1.28)
Liu et al. (38)	China	Cross-sectional study	1,251	Harmonized criteria	2 vs. ≤1 ≥3 vs. ≤1	1.36 (0.95, 1.96) 1.75 (1.19, 2.57)
Wu et al. (39)	China	Cross-sectional study	13,358	IDF	2 vs. 1 3 vs. 1 ≥4 vs. 1	1.18 (1.05, 1.32) 1.44 (1.24, 1.67) 1.52 (1.26, 1.83)
Akter et al. (45)	Bangladeshi	Cross-sectional study	1,219	NCEP ATP III	2 vs. ≤1 3 vs. ≤1 ≥4 vs. ≤1	1.10 (0.70, 1.73) 1.26 (0.78, 2.05) 1.65 (1.00, 2.72)
Cho et al. (20)	Korea	Cross-sectional study	892	NCEP ATP III	≥1 vs. Nulliparous	1.04 (0.92, 1.17)
Mousavi et al. (44)	Iran	Cross-sectional study	6,331	NCEP ATP III	per 1 live birth	1.02 (0.98, 1.05)
Gunderson et al. (41)	United States	Cohort Study	1,451	NCEP ATP III	1 vs. Nulliparous ≥2 vs. Nulliparous	1.33 (0.93, 1.90) 1.62 (1.16, 2.26)
Al-barwani et al. (46)	Oman	Cross-sectional study	392	IDF	1–3 vs. Nulliparous 4–6 vs. Nulliparous >6 vs. Nulliparous	1.70 (0.50, 5.90) 1.80 (0.50, 6.30) 3.00 (1.10, 9.30)
Cohen et al. (42)	United States	Cross-sectional study	4,699	NCEP ATP III	per 1 live birth	1.13 (1.06, 1.20)
Lao et al. (40)	China	Cohort Study	7,352	IDF	per 1 live birth	1.10 (1.03, 1.18)

*IDF, International Diabetes Federation; MetS, metabolic syndrome; NCEP ATP III, National Cholesterol Education Program Adult Treatment Panel III.*

Majorities of the included articles were considered for the important confounder adjustments in their primary analyses, for instance, age (*n* = 15), smoking status (*n* = 11), and body mass index (*n* = 8). In addition, fewer was adjusted for age at menarche (*n* = 3), age at first pregnancy (*n* = 3), menopause status (*n* = 3), and hip circumference (*n* = 2) (Table 2).

**TABLE 2:** Adjustment potential confounders of included studies.

References	Adjustment for potential confounders in the primary analysis
Shi et al. (19)	Age, smoking, drinking, exercise, education first-degree relatives of patients with diabetes, pregnancy losses, age at menarche, duration of reproductive years, exercise, BMI, hip circumference
Xie et al. (18)	Age, HbA1c, TC, number of live-birth pregnancies, hip circumference, DBP
Yao et al. (10)	Age, postmenopausal status, marital status, current smoking, alcohol use, oral contraceptive use, income, physical activity, education level
Lee et al. (21)	Age, smoking, drinking, exercise, income, education, breast feeding, oral contraceptive use, age at menarche
Vladutiu et al. (17)	Age, Hispanic/Latino background, income, education, marital status, nativity, smoking, physical activity, menopausal status, oral contraceptive use, hormone therapy, field center
Moradi et al. (43)	Age, age at first pregnancy, duration of lactation, number of pregnancies, histories of DM and hypertension
Liu et al. (38)	Age, education, marital status, ever smoking, ever drinking, physical activity, BMI, family history of CVD
Wu et al. (39)	Age, education, marital status, smoking status, drinking, physical activity, menopause status, abortion, BMI, use of contraceptives, ever use of hormone replacement therapy
Akter et al. (45)	Age, BMI, marital status, tobacco, use of contraceptives, education, age at first pregnancy
Cho et al. (20)	Age, BMI, marital status, smoking, education level, income, lifestyle, alcohol intake, exercise
Mousavi et al. (44)	Age, education, residence, family income, currently employed, BMI, reproductive, smoking, physical activity
Gunderson et al. (41)	Age, race, BMI, education, smoking
Al-barwani et al. (46)	Age
Cohen et al. (42)	Age, race, income, education, the interaction between non-Hispanic black race, parity
Lao et al. (40)	Age, education, physical activity, BMI, occupation, income, drinking, marital status, smoking, age at menarche, age at menopause, age at first pregnancy, use of contraceptive pills

*BMI, body mass index; CVD, cardiovascular disease; DM, diabetes mellitus; DBP, diastolic blood pressure; TC, total cholesterol; HbA1c, Glycosylated Hemoglobin, Type A1C.*

The average score for the thirteen cross-sectional studies was 8.917, with a range from 7 to 9, which was considered moderate quality. For two cohort studies, both of them had the maximum number of stars in two fields, which were deemed as high quality. The quality assessment of included articles is shown in Supplementary Tables 1, 2.

### Ever Parity vs. Nulliparous

**FIGURE 2:**
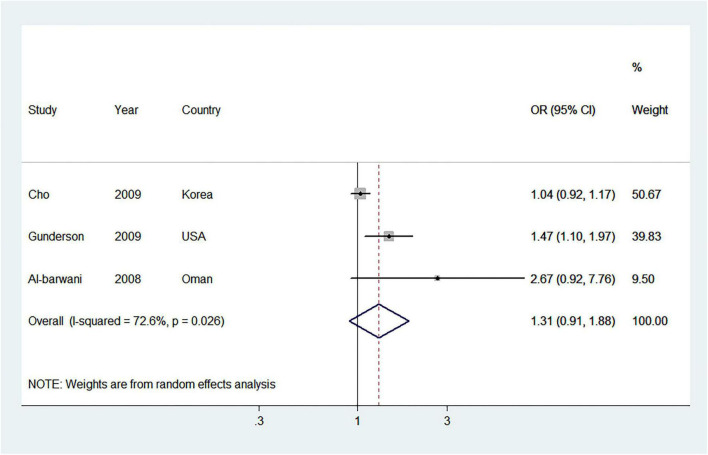
Forest plot (random-effects model) of ever parity and metabolic syndrome risk, comparing the nulliparous. Squares indicate study-specific odds ratio (OR), where the size of the square reflects the study-specific statistical weight; horizontal lines indicate the 95% CI; diamonds denote the summary OR with 95% CI.

Three studies (20, 41, 46) reported the association between ever parity and MetS risk. As compared with the nulliparous, the pooled OR of MetS for the ever parity was 1.31 (95% CI = 0.91–1.88) with moderate heterogeneity (*I*^2^ = 72.6%) (Figure 2). We observed no publication bias for Begg’s test (*P* = 1.000) and Egger’s test (*P* = 0.228).

### Highest vs. Lowest Parity

**FIGURE 3:**
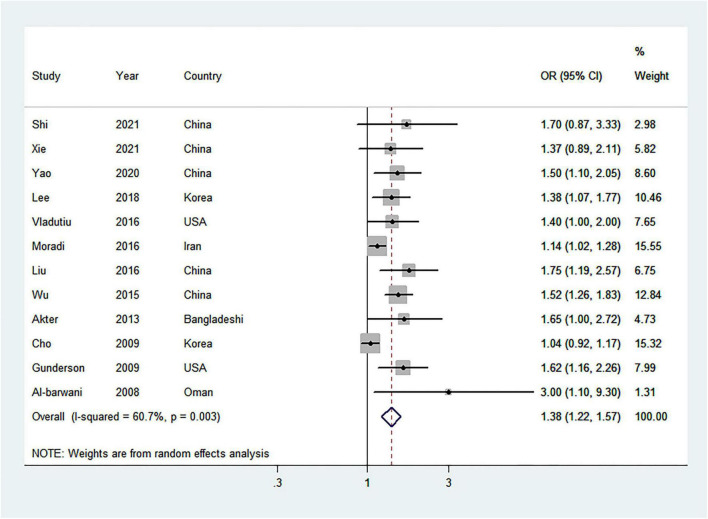
Forest plot (random-effects model) of the highest parity number and metabolic syndrome risk, comparing the lowest parity number. Squares indicate study-specific odds ratio (OR), where the size of the square reflects the study-specific statistical weight; horizontal lines indicate the 95% CI; diamonds denote the summary OR with 95% CI.

After meta-analyzing twelve studies (10, 17–21, 38, 39, 41, 43, 45, 46), including 43,713 women, it was used to evaluate the association between the highest vs. lowest analysis of parity number and the risk of MetS. As compared with the lowest parity number, the pooled OR of MetS for the highest parity number was 1.38 (95% CI = 1.22–1.57) with moderate heterogeneity (*I*^2^ = 60.7%) (Figure 3). No publication bias was found from Begg’s test (*P* = 0.373), but Egger’s test (*P* = 0.001) and the visual funnel plot found significant publication bias (Supplementary Figure 1). However, the overall effect estimate was 1.20 (95% CI = 1.06–1.36) by the use of trim-and-fill methods, indicating that the findings were unaffected by publication bias.

### Dose-Response, Subgroup, and Sensitivity Analysis

**FIGURE 4:**
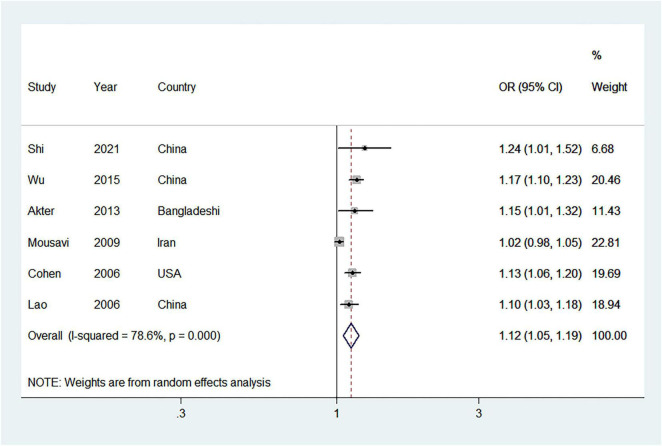
Forest plot (random-effects model) for the linear dose-response relationship between parity and metabolic syndrome (per 1 live birth). Squares indicate study-specific odds ratio (OR), where the size of the square reflects the study-specific statistical weight; horizontal lines indicate the 95% CI; diamonds denote the summary OR with 95% CI.

A total of 6 studies (19, 39, 40, 42, 44, 45) were incorporated into the linear dose-response relationship of parity and MetS risk. The pooled OR per 1 live birth from parous females was 1.12 (95% CI = 1.05–1.19) and heterogeneity between studies was high (*I*^2^ = 78.6%) (Figure 4). No publication bias was found (Begg’s *P* = 1.000, Egger’s *P* = 0.132, and the visual funnel plot) (Supplementary Figure 2).

Most results of subgroup analysis were consistent with the main findings (Table 3). As with the highest vs. lowest parity, the point estimation per 1 live birth for non-Asia was higher than in Asia. Eligible studies adjusting for oral contraceptive use and alcohol drinking appeared to have a much higher effect estimate than those not adjusting. Additionally, no evidence of heterogeneity between subgroups was detected by meta-regression analyses.

**TABLE 3:** Subgroup analyses for parity and risk of metabolic syndrome (highest vs. lowest and dose-response analysis).

	Highest vs. lowest					Dose-response analysis				
	No. of study	OR (95%CI)	*I^2^ (%)*	*P* ^1^	*P* ^2^	No. of study	OR (95%CI)	*I^2^ (%)*	*P* ^1^	*P* ^2^
**Overall**	12	1.38 (1.22, 1.57)	60.70	<0.01		6	1.12 (1.05, 1.19)	78.60	<0.01	
**Subgroup analyses**										
**Region**					0.229					0.664
Asia	8	1.32 (1.15, 1.52)	66.00	<0.01		4	1.11 (1.02, 1.21)	84.90	<0.01	
Non-Asia	4	1.58 (1.28, 1.95)	0.0	0.593		2	1.13 (1.07, 1.20)	0.0	0.816	
**Study design**					0.475					0.845
Cross-sectional study	11	1.36 (1.19, 1.55)	60.60	<0.01		5	1.12 (1.04, 1.21)	82.60	<0.01	
Cohort study	1	1.62 (1.16, 2.26)	NA	NA		1	1.10 (1.03, 1.18)	NA	NA	
**Study population** *					0.464					0.404
<Mean	7	1.35 (1.12, 1.62)	66.10	<0.01		3	1.14 (1.08, 1.20)	0.0	0.690	
≥Mean	5	1.46 (1.29, 1.64)	0.0	0.970		3	1.09 (1.00, 1.19)	88.70	<0.01	
**Menopausal status**					0.195					0.761
Post-menopausal	3	1.23 (0.94, 1.59)	64.00	0.062		2	1.12 (1.03, 1.22)	15.90	0.276	
Non- postmenopausal	9	1.45 (1.25, 1.67)	49.80	0.043		4	1.11 (1.02, 1.20)	85.80	<0.01	
**Diagnostic criteria**					0.274					0.955
NCEP ATP III	6	1.26 (1.08, 1.46)	59.00	0.032		3	1.09 (1.00, 1.18)	79.70	<0.01	
IDF	2	1.73 (1.03, 2.91)	33.90	0.219		2	1.14 (1.07, 1.21)	47.10	0.169	
**Adjust body mass index**					0.995					0.833
Yes	6	1.46 (1.15, 1.85)	75.50	<0.01		5	1.11 (1.03, 1.20)	80.90	<0.01	
No	6	1.31 (1.14, 1.52)	33.70	0.184		1	1.13 (1.06, 1.20)	NA	NA	
**Adjust alcohol drinking**					0.945					0.325
Yes	6	1.40 (1.14, 1.71)	73.50	<0.01		3	1.14 (1.09, 1.20)	19.80	0.287	
No	6	1.39 (1.14, 1.68)	45.20	0.104		3	1.09 (1.00, 1.18)	79.70	<0.01	
**Adjust cigarette smoking**					0.823					0.833
Yes	9	1.43 (1.22, 1.68)	64.40	<0.01		5	1.11 (1.03, 1.20)	80.90	<0.01	
No	3	1.30 (0.95, 1.78)	45.90	0.157		1	1.13 (1.06, 1.20)	NA	NA	
**Adjust oral contraceptive use**					0.266					0.405
Yes	5	1.47 (1.31, 1.67)	0.0	0.956		3	1.14 (1.10, 1.19)	0.0	0.387	
No	7	1.32 (1.11, 1.57)	63.30	0.012		3	1.09 (0.99, 1.21)	81.20	<0.01	
**Adjust age at first pregnancy**					0.426					0.946
Yes	2	1.26 (0.91, 1.74)	49.90	0.158		2	1.11 (1.04, 1.18)	0.0	0.562	
No	10	1.43 (1.22, 1.68)	62.50	<0.01		4	1.12 (1.02, 1.22)	86.40	<0.01	

*CI, confidence interval; IDF, International Diabetes Federation; NA, not applicable; NCEP ATP III, National Cholesterol Education Program Adult Treatment Panel III; OR, odds ratio.*

*^1^P-Value for heterogeneity within each subgroup.*

*^2^P-Value for heterogeneity between subgroups with meta-regression analysis.*

*The mean study population for the analysis of parity (highest vs. lowest) is 3,643; the mean study population for the dose-response analysis of parity is 5,623.

In order to determine the robustness of the results, we performed sensitivity analyses. Our sensitivity analysis of highest vs. lowest parity showed that the OR for MetS ranged from 1.36 (95% CI = 1.19–1.55, *I*^2^ = 56.0%) when Wu et al. (39) were removed to 1.44 (95% CI = 1.24–1.68, *I*^2^ = 59.9%) when Moradi et al. (43) were removed (Supplementary Figure 3). Similarly, in the dose-response analysis, we also explore the stability of the pooled OR between parity and MetS per 1 live birth from parous females; the pooled OR for MetS ranged from 1.10 (95% CI = 1.03–1.17, *I*^2^ = 70.8%) when Wu et al. (39) were removed to 1.12 (95% CI = 1.04–1.21, *I*^2^ = 82.6%) when Lao et al. (40) were removed (Supplementary Figure 4).

## Discussion

In this present meta-analysis, we comprehensively evaluated the available evidence on the relationship between parity and MetS among 62,095 women. Ever parity was not associated with MetS risk when compared with the nulliparous. However, in the dose-response analyses, with a 12% increase in risk of MetS per 1 live birth from parous females.

Although a significant difference between regions in the subgroup analysis (1.13 for non-Asia, 1.11 for Asia) was not found, region differences still should be considered. For example, when investigating 17,048 adults from 2011 to 2016 in the US, it was found that the weighted prevalence of MetS was 37.4% (47), whereas Farmanfarma et al. (48) found that the prevalence of MetS was 21.8% based on 761 individuals from 2009 to 2017 in Iran. Even so, we still have to consider the risk of MetS associated with non-Asian regions that are less commonly assessed, such as Europe and North America. Furthermore, different fertility policies will also affect the research results. These policies will create sociodemographic confounding factors in the process of implementation (49).

In the subgroup analysis stratified by diagnostic criteria, compared to the results of the National Cholesterol Education Program Adult Treatment Panel III (NCEP ATP III) criteria, the risk for MetS from the International Diabetes Federation (IDF) criteria was detected to be substantially higher in parous females. The prevalence of MetS depends not only on regional, but also depends on the diagnostic criteria. Epidemiological studies have also shown that prevalence was 45.5% through the use of the IDF criteria, but 24.3% according to the NCEP ATP III criteria in Tunisia (50). In addition, the definition of abdominal obesity was different by various criteria and the IDF criteria have a lower waist circumference cutoff value than the NCEP ATP III criteria (≥80 vs. ≥88 cm) (1). Consequently, more participants were diagnosed with MetS by the IDF standard, which could be a source of heterogeneity in the results.

Further concerns regarding parity and MetS risk are menopausal status. A previous cross-sectional study indicated that the harmful effects of parity on MetS were more evident in postmenopausal women (18). Our meta-analysis confirmed that the pooled effect estimate of MetS in postmenopausal women was higher than in non-postmenopausal women. This could mean that the harmful effects of parity are usually in the long term, which can be observed after the postmenopausal period due tothe significant changes in hormones around menopause (16). Another explanation could be the MetS prevalence in different menopausal women. For instance, a retrospective study of 958 women indicated that the MetS prevalence was 22.2% in postmenopausal women and 9.4% in premenopausal women (51). The two included observational studies reported a positive correlation between parity and MetS in postmenopausal women (18, 21). These phenomena indicated that menopausal status was an important risk factor for the incidence of MetS (18). Due to the limited included eligible relevant studies, we could not be excluded the possibility of chance findings in our subgroup analysis.

Several potential biological mechanisms might be explanations for the adverse effects of parity and MetS risk. First, estrogen levels change during pregnancy and women who give birth have about 22% less estrogen than women who do not give birth (16). In addition, the reduction of estrogen exposure will cause the disorder of lipid metabolism, hypertension, hyperglycemia, and other components of MetS (52, 53), which lead to the occurrence of MetS. Second, pregnancy can cause a series of physiological function changes, including but not limited to insulin resistance, abnormal lipid metabolism, hypertension, and central obesity (54–57). Insulin resistance always appears simultaneously with other metabolic-related diseases, which play a crucial role in the development of MetS (15). Hence, repeated pregnancy may have long-term effects on the health of parous females.

The strengths of our meta-analysis should be emphasized. First, this is the first quantitative dose-response meta-analysis to investigate the association between parity and MetS risk. Second, we systematically searched the literature database, eligible relevant articles were identified, and evaluated the risk of bias for the primary studies. Third, for the study characteristics and the adjustment of potential confounders, we conducted subgroup and meta-regression analyses to explore sources of heterogeneity evidence. In addition, sensitivity analyses were carried out to determine the robustness of the findings.

The disadvantage of our meta-analysis also should be outlined. First, most included studies were cross-sectional studies, which could only reflect the situation at a certain time point; the selection and recall bias were inevitable. However, recall bias and misclassification regarding parity numbers seem unlikely in our analysis. In addition, the observed risk estimate from cross-sectional studies (OR = 1.12, 95% CI = 1.04–1.21) was similar to that from cohort studies (OR = 1.10, 95% CI = 1.03–1.18). This matter could not influence the findings. Second, although most of the confounding factors have been adjusted in primary studies, some residual confounding factors associated with parity, such as menarche age, menstruation, menopause, and other reproductive factors, have not been controlled in these original studies. Therefore, unknown or uncontrolled confounders may bias the pooled risk estimate. Third, the definition of MetS was varied among these articles, which could affect the interpretation of the results. For example, when comparing the highest vs. lowest parity, an observational study in Oman indicated that the results were different when using different definitions of MetS (OR = 3.0 for the IDF vs. OR = 1.9 for the NCEP ATP III) (46). The main variability between these two definitions is that the IDF recommended abdominal obesity as a prerequisite for the diagnosis of MetS and the waist circumference cutoff point was lower than the NCEP ATP III recommendations (56). Therefore, it is emphasized that standard definitions need widespread adoption to promote comparability across studies in the future. Fourth, openly published literature was only searched and analyzed, while other non-English literature and gray literature that meet our inclusion criteria may be neglected. Fifth, this meta-analysis did not evaluate whether the increased risk of MetS was transient or long term because the studies that we included did not investigate the transient or long-lasting effects of parity numbers on MetS. These effects need to be explored in detail in the future. In addition, due to the limited included studies (*n* = 3), we failed to investigate non-linear associations between parity and MetS risk. Finally, we found a publication bias for the highest vs. lowest parity and MetS risk. However, the pooled effect estimate was unaltered by trim-and-fill analysis, indicating that the publication bias was negligible.

## Conclusion

Our present meta-analysis reveals valuable evidence that an increased parity number may be associated with an increased risk of MetS. A sufficient number of large-scale, high-quality prospective studies are needed to validate our results.

## Data Availability Statement

The original contributions presented in this study are included in the article/Supplementary Material, further inquiries can be directed to the corresponding authors.

## Author Contributions

M-HS, Z-YW, Q-JW, and Y-HZ conceived the study, contributed to the design, and interpreted the data. M-HS, Z-YW, and Q-JW collected the data. M-HS and Z-YW cleaned the data, checked the discrepancy, analyzed the data, and contributed equally to this study. M-HS, Z-YW, RW, CG, J-LY, Y-JC, Q-JW, and Y-HZ interpreted the data, read the manuscript, and approved the final version. All authors have contributed to the articles and approved the submitted version of the manuscript.

## Conflict of Interest

The authors declare that the research was conducted in the absence of any commercial or financial relationships that could be construed as a potential conflict of interest.

## Publisher’s Note

All claims expressed in this article are solely those of the authors and do not necessarily represent those of their affiliated organizations, or those of the publisher, the editors and the reviewers. Any product that may be evaluated in this article, or claim that may be made by its manufacturer, is not guaranteed or endorsed by the publisher.
