# Supplementary material for: Parity and Metabolic Syndrome Risk: A Systematic Review and Meta-Analysis of 15 Observational Studies With 62,095 Women
Source: Front Med (Lausanne). 2022 Jul 12;9:926944. doi: 10.3389/fmed.2022.926944 (PMC9314745; doi:10.3389/fmed.2022.926944)
Supplement: Supplementary file 1 [file Data_Sheet_1.docx]

Supplementary Material

**Supplementary Table 1. Methodological quality of cross-sectional studies included in the meta-analysis**

| **First author,**  **reference** | Shi,  (19) | Xie,  (18) | Yao,  (10) | Lee,  (21) | Vladutiu,  (17) | Moradi,  (43) | Liu,  (38) | Wu,  (39) | Akter,  (45) | Cho,  (20) | Mousavi,  (44) | Al-barwani,  (46) | Cohen,  (42) |
| --- | --- | --- | --- | --- | --- | --- | --- | --- | --- | --- | --- | --- | --- |
| **1. Was the research question or objective in this paper clearly stated?** | Yes | Yes | Yes | Yes | Yes | Yes | Yes | Yes | Yes | Yes | Yes | Yes | Yes |
| **2. Was the study population clearly specified and defined?** | Yes | Yes | Yes | Yes | Yes | Yes | Yes | Yes | Yes | Yes | Yes | Yes | Yes |
| **3. Was the participation rate of eligible persons at least 50%?** | No | Yes | Yes | No | Yes | Yes | Yes | Yes | Yes | Yes | Yes | Yes | Yes |
| **4. Were all the subjects selected or recruited from the same or similar populations (including the same time period)?** | Yes | Yes | Yes | Yes | Yes | Yes | Yes | Yes | Yes | Yes | Yes | Yes | Yes |
| **5. Were inclusion and exclusion criteria for being in the study prespecified and applied uniformly to all participants?** | Yes | Yes | Yes | Yes | Yes | Yes | Yes | Yes | Yes | Yes | Yes | Yes | Yes |
| **6. Was a sample size justification, power description, or variance and effect estimates provided?** | No | No | No | No | No | No | No | No | No | No | No | No | No |
| **7. For exposures that can vary in amount or level, did the study examine different levels of the exposure as related to the outcome?** | Yes | Yes | Yes | Yes | Yes | No | Yes | Yes | Yes | No | No | Yes | Yes |
| **8. Were the exposure measures (independent variables) clearly defined, valid, reliable, and implemented consistently across all study participants?** | No | No | Yes | Yes | Yes | No | Yes | Yes | Yes | Yes | Yes | No | Yes |
| **9. Was the exposure(s) assessed more than once over time?** | No | No | No | No | No | No | No | No | No | No | No | No | No |
| **10. Were the outcome measures (dependent variables) clearly defined, valid, reliable, and implemented consistently across all study participants?** | Yes | Yes | Yes | Yes | Yes | Yes | Yes | Yes | Yes | Yes | Yes | Yes | Yes |
| **11. Were key potential confounding variables measured and adjusted statistically for their impact on the relationship between exposure(s) and outcome(s)?** | Yes | Yes | Yes | Yes | Yes | Yes | Yes | Yes | Yes | Yes | Yes | No | Yes |
| **Total** | 7 | 8 | 9 | 8 | 9 | 7 | 9 | 9 | 9 | 8 | 8 | 7 | 9 |

The definition/explanation for each column is available from National Institutes of Health Quality assessment Tool for Observational Cohort and Cross-Sectional Studies.

(

" [https://www.nhlbi.nih.gov/health-topics/study-quality-assessment-tool).]("https://www.nhlbi.nih.gov/health-topics/study-quality-assessment-tool)

["]("https://www.nhlbi.nih.gov/health-topics/study-quality-assessment-tool) **[Supplementary Table 2. Methodological quality of cohort studies included in the meta-analysis]("https://www.nhlbi.nih.gov/health-topics/study-quality-assessment-tool) ^[a]("https://www.nhlbi.nih.gov/health-topics/study-quality-assessment-tool)^**

|  | **[Selection]("https://www.nhlbi.nih.gov/health-topics/study-quality-assessment-tool)** | | | | **[Comparability]("https://www.nhlbi.nih.gov/health-topics/study-quality-assessment-tool)** |  | **[Outcome]("https://www.nhlbi.nih.gov/health-topics/study-quality-assessment-tool)** |  |  |
| --- | --- | --- | --- | --- | --- | --- | --- | --- | --- |
| **[First author,]("https://www.nhlbi.nih.gov/health-topics/study-quality-assessment-tool)**  **[reference,]("https://www.nhlbi.nih.gov/health-topics/study-quality-assessment-tool)**  **[publication year]("https://www.nhlbi.nih.gov/health-topics/study-quality-assessment-tool)** | **[Representativeness]("https://www.nhlbi.nih.gov/health-topics/study-quality-assessment-tool)**  **[of the exposed cohort]("https://www.nhlbi.nih.gov/health-topics/study-quality-assessment-tool)** | **[Selection of the unexposed]("https://www.nhlbi.nih.gov/health-topics/study-quality-assessment-tool)**  **[cohort]("https://www.nhlbi.nih.gov/health-topics/study-quality-assessment-tool)** | **[Ascertainment]("https://www.nhlbi.nih.gov/health-topics/study-quality-assessment-tool)**  **[of exposure]("https://www.nhlbi.nih.gov/health-topics/study-quality-assessment-tool)** | **[Outcome of interest]("https://www.nhlbi.nih.gov/health-topics/study-quality-assessment-tool)**  **[not present]("https://www.nhlbi.nih.gov/health-topics/study-quality-assessment-tool)**  **[at start of study]("https://www.nhlbi.nih.gov/health-topics/study-quality-assessment-tool)** | **[Control for]("https://www.nhlbi.nih.gov/health-topics/study-quality-assessment-tool)**  **[important factor or additional factor]("https://www.nhlbi.nih.gov/health-topics/study-quality-assessment-tool) ^[b]("https://www.nhlbi.nih.gov/health-topics/study-quality-assessment-tool)^** | **[Assessment of outcome]("https://www.nhlbi.nih.gov/health-topics/study-quality-assessment-tool)** | **[Follow-up]("https://www.nhlbi.nih.gov/health-topics/study-quality-assessment-tool)**  **[long enough for outcomes]("https://www.nhlbi.nih.gov/health-topics/study-quality-assessment-tool)**  **[to occur]("https://www.nhlbi.nih.gov/health-topics/study-quality-assessment-tool) ^[c]("https://www.nhlbi.nih.gov/health-topics/study-quality-assessment-tool)^** | **[Adequacy of]("https://www.nhlbi.nih.gov/health-topics/study-quality-assessment-tool)**  **[follow-up]("https://www.nhlbi.nih.gov/health-topics/study-quality-assessment-tool)**  **[of cohorts]("https://www.nhlbi.nih.gov/health-topics/study-quality-assessment-tool) ^[d]("https://www.nhlbi.nih.gov/health-topics/study-quality-assessment-tool)^** | **[Risk of bias]("https://www.nhlbi.nih.gov/health-topics/study-quality-assessment-tool) ^[e]("https://www.nhlbi.nih.gov/health-topics/study-quality-assessment-tool)^** |
| [Gunderson et al. (41), 2009]("https://www.nhlbi.nih.gov/health-topics/study-quality-assessment-tool) | [*]("https://www.nhlbi.nih.gov/health-topics/study-quality-assessment-tool) | [*]("https://www.nhlbi.nih.gov/health-topics/study-quality-assessment-tool) | [*]("https://www.nhlbi.nih.gov/health-topics/study-quality-assessment-tool) | [*]("https://www.nhlbi.nih.gov/health-topics/study-quality-assessment-tool) | [**]("https://www.nhlbi.nih.gov/health-topics/study-quality-assessment-tool) | [*]("https://www.nhlbi.nih.gov/health-topics/study-quality-assessment-tool) | [*]("https://www.nhlbi.nih.gov/health-topics/study-quality-assessment-tool) | [-]("https://www.nhlbi.nih.gov/health-topics/study-quality-assessment-tool) | [Low risk]("https://www.nhlbi.nih.gov/health-topics/study-quality-assessment-tool) |
| [Lao et al. (40), 2006]("https://www.nhlbi.nih.gov/health-topics/study-quality-assessment-tool) | [*]("https://www.nhlbi.nih.gov/health-topics/study-quality-assessment-tool) | [*]("https://www.nhlbi.nih.gov/health-topics/study-quality-assessment-tool) | [*]("https://www.nhlbi.nih.gov/health-topics/study-quality-assessment-tool) | [*]("https://www.nhlbi.nih.gov/health-topics/study-quality-assessment-tool) | [**]("https://www.nhlbi.nih.gov/health-topics/study-quality-assessment-tool) | [*]("https://www.nhlbi.nih.gov/health-topics/study-quality-assessment-tool) | [-]("https://www.nhlbi.nih.gov/health-topics/study-quality-assessment-tool) | [-]("https://www.nhlbi.nih.gov/health-topics/study-quality-assessment-tool) | [Low risk]("https://www.nhlbi.nih.gov/health-topics/study-quality-assessment-tool) |

[The definition/explanation of the Newcastle-Ottawa Scale in each column is available from (]("https://www.nhlbi.nih.gov/health-topics/study-quality-assessment-tool)<http://www.ohri.ca/programs/clinical_epidemiology/oxford.asp>[).]("https://www.nhlbi.nih.gov/health-topics/study-quality-assessment-tool)

^[a]("https://www.nhlbi.nih.gov/health-topics/study-quality-assessment-tool)^ [A study could be awarded a maximum of one star for each item, except control for important or additional factors.]("https://www.nhlbi.nih.gov/health-topics/study-quality-assessment-tool)

^[b]("https://www.nhlbi.nih.gov/health-topics/study-quality-assessment-tool)^ [A maximum of two stars could be awarded for this item. Studies that controlled for age received one star whereas studies that controlled for other important confounders, such as body mass index, income, or physical activity received an additional star.]("https://www.nhlbi.nih.gov/health-topics/study-quality-assessment-tool)

^[c]("https://www.nhlbi.nih.gov/health-topics/study-quality-assessment-tool)^ [A cohort study with follow-up time > 24 months was assigned one star.]("https://www.nhlbi.nih.gov/health-topics/study-quality-assessment-tool)

^[d]("https://www.nhlbi.nih.gov/health-topics/study-quality-assessment-tool)^ [A cohort study with a follow-up rate > 75% was assigned one star.]("https://www.nhlbi.nih.gov/health-topics/study-quality-assessment-tool)

^[e]("https://www.nhlbi.nih.gov/health-topics/study-quality-assessment-tool)^ [Studies that obtained full scores at least two domains were considered to have a low risk of bias, other situations were considered as high risk.]("https://www.nhlbi.nih.gov/health-topics/study-quality-assessment-tool)


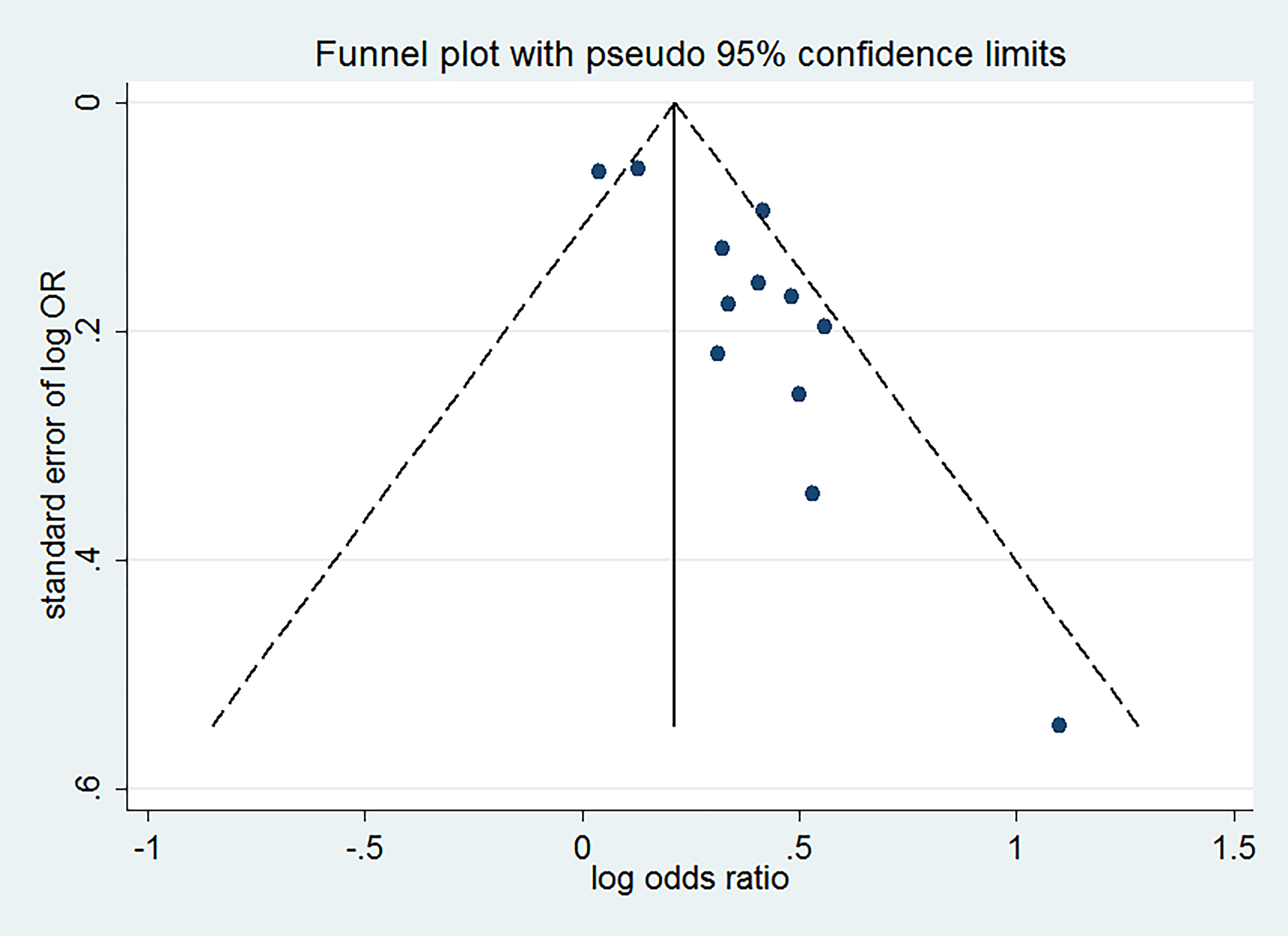


**Supplementary Figure 1**. Funnel plots for parity number (highest versus lowest) and risk of MetS.


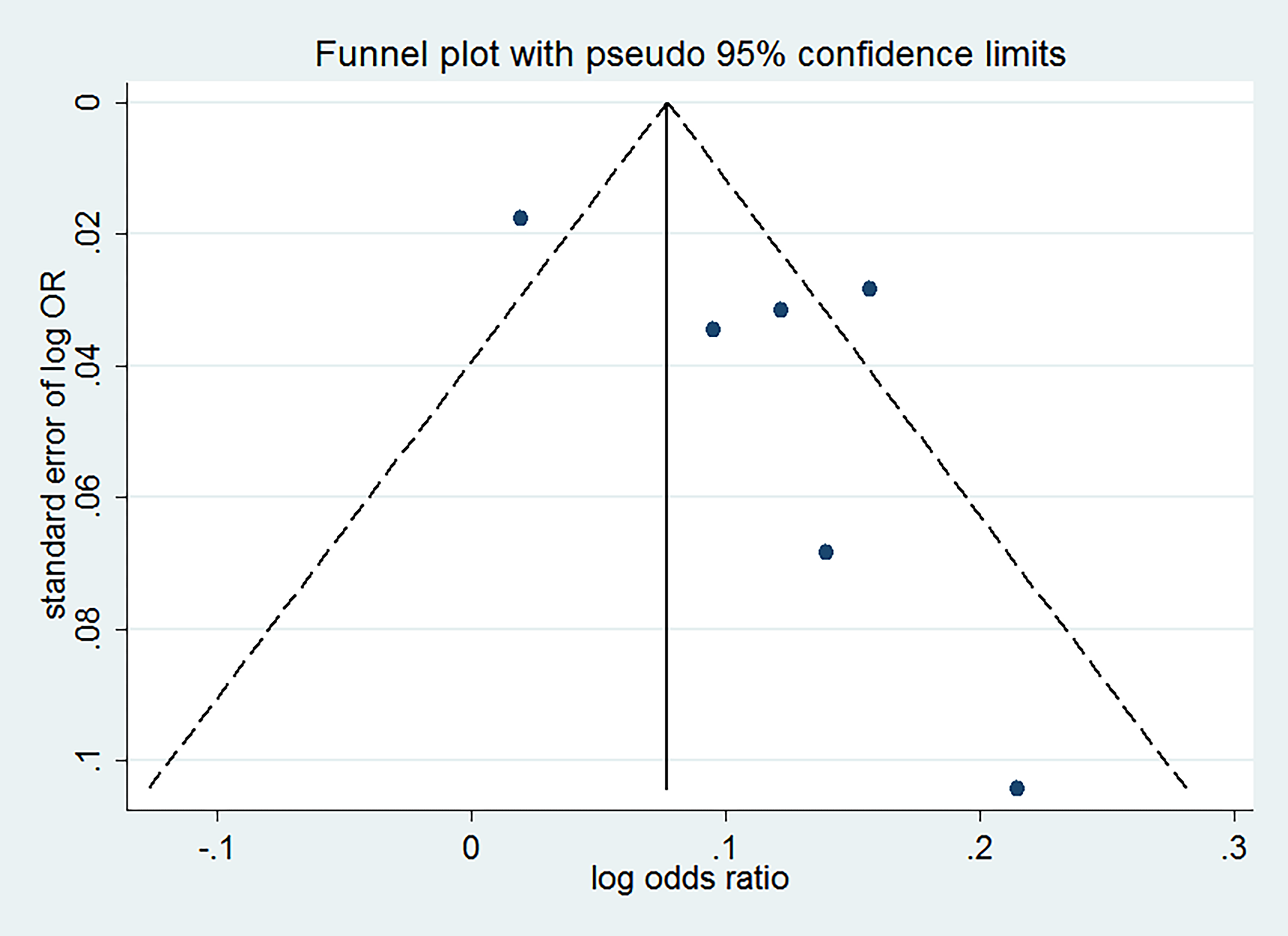


**Supplementary Figure 2**. Funnel plots for parity number (per 1 live birth) and risk of MetS.


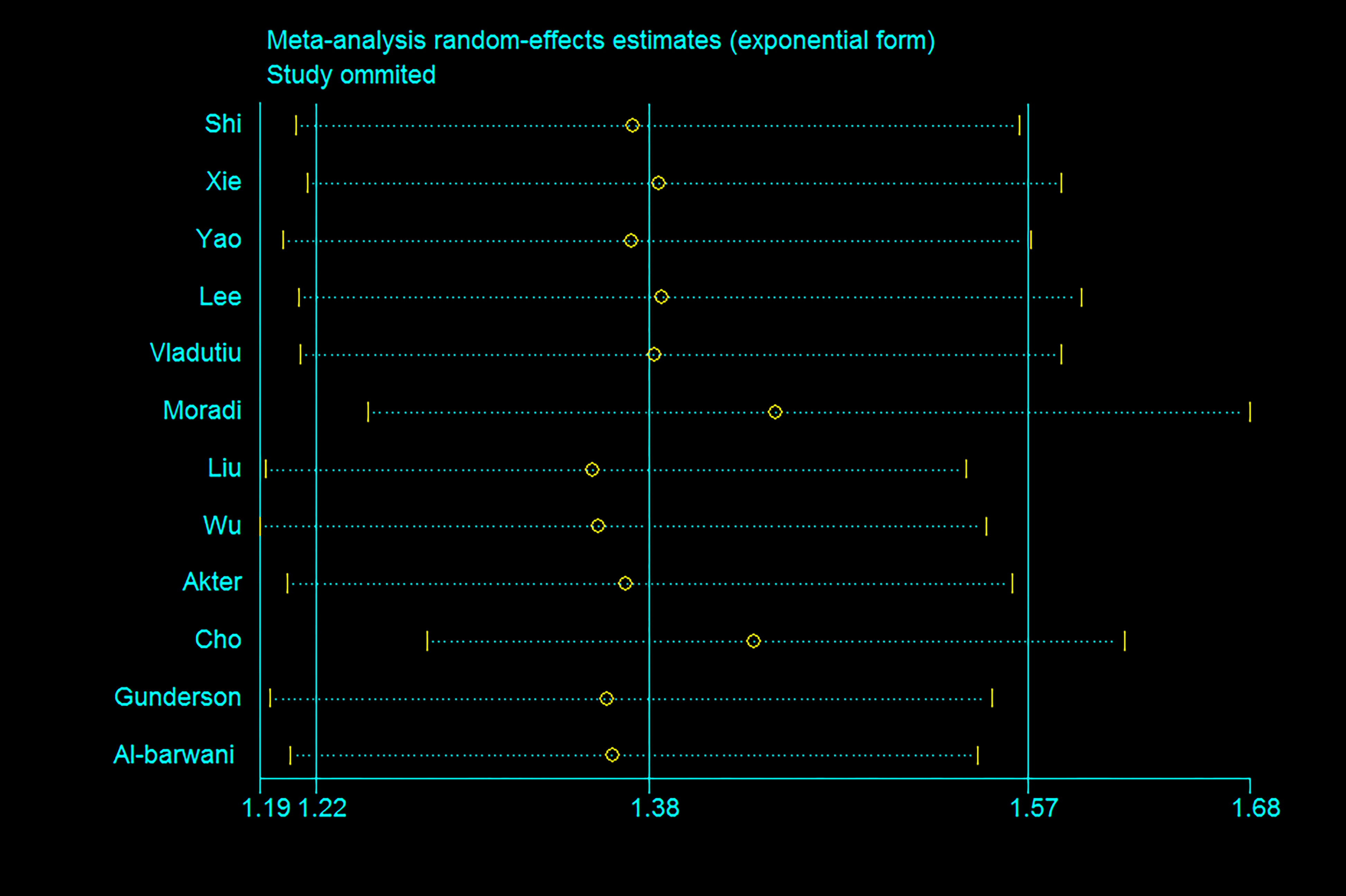


**Supplementary Figure 3**. Sensitivity analysis of meta-analysis of parity number (highest versus lowest) and MetS risk by excluding each study in sequence.


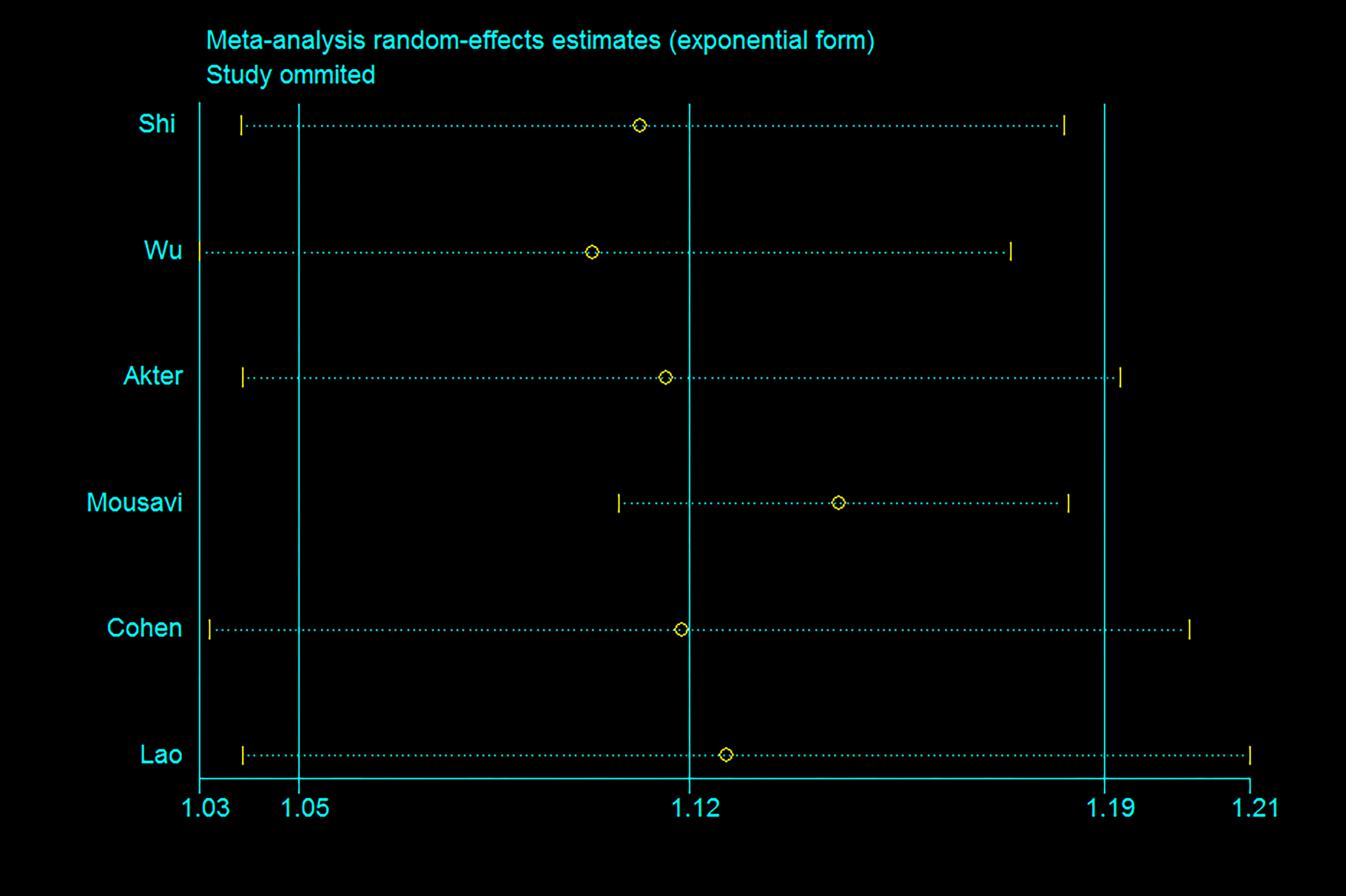


**Supplementary Figure 4**. Sensitivity analysis of meta-analysis of parity number (per 1 live birth) and MetS risk by excluding each study in sequence.
